# A catalog of bird specimens associated with Prince Maximilian of Wied-Neuwied and potential type material in the natural history collection in Wiesbaden

**DOI:** 10.3897/zookeys.353.4198

**Published:** 2013-11-20

**Authors:** Dorothee Hoffmann, Fritz Geller-Grimm

**Affiliations:** 1 Museum Wiesbaden – Hessisches Landesmuseum für Kunst und Natur, Naturhistorische Sammlungen, Friedrich-Ebert-Allee 2, 65185 Wiesbaden, Germany

**Keywords:** Aves, types, museum specimens, Brazil, Mata Atlântica

## Abstract

Bird specimens collected by 19^th^ century explorer and ornithologist Prince Maximilian of Wied-Neuwied form one of the foundation collections of the American Museum of Natural History in New York. However, parts of his collection remained in Germany and came to the Museum Wiesbaden. Since Wied described numerous new species without designating types, some of these specimens might be type material. Here we present a catalog of the 30 Wiesbaden specimens associated with him and discuss their potential type status. We conclude that 17 individuals in 11 species are potential type specimens that should be considered in future taxonomic work.

## Introduction

The natural history collection at the Hessian state museum in Wiesbaden (MWNH) owns several specimens that either originate from the collection of Prince Maximilian of Wied-Neuwied or were described by him as new species. In the early 19^th^ century Wied was among the first explorers to travel to Brazil and the especially diverse ecoregion Mata Atlântica, where he collected large numbers of ethnographic objects, plants and animals. In 1870 part of the Wied collection comprising 4,000 birds, 600 mammals and 2,000 fish and reptiles was purchased by the American Museum of Natural History in New York (AMNH) and constitutes the cornerstone of its scientific collection (Myers 2000).

Besides mammals, reptiles, and amphibians, Wied described 160 species and subspecies of birds. The names of more than 50 of these taxa are still valid today (Encyclopedia of Life). Potential type specimens of the majority (ca. 120) could be recognized at the AMNH. At least some of the specimens presented here came to the collection in Wiesbaden in Wied’s lifetime. The AMNH type catalogs published to date (Allen 1889, 1891; Greenway 1973, 1978, 1987; LeCroy and Sloss 2000; LeCroy 2003, 2005, 2012) do not offer any information on material in Wiesbaden. However, we must assume that more material exists outside the AMNH. To begin with, the corresponding types of 40 taxa are not in the AMNH collection, and furthermore, Wied himself recorded only limited information on the material he studied and, as was common practice at that time, did not designate types. A systematic research for more material is still outstanding, and since at that time it was usual to barter, trade and give away undocumented specimens, some surprising discoveries may still be expected. Berger (1995) reports later divestitures of material by the AMNH to other institutions, like the Smithsonian in Washington, D.C. De Avila-Pires (1965) and Engländer (1995) mention other collections; for instance besides New York, mammal types have also been deposited in Leiden and Paris.

Like at the AMNH, in Wiesbaden as well many of the originally mounted specimens were later dismounted and added to the study skin collection. Most of the series of the main Wied collection at the AMNH still bear their original labels, but not every individual specimen has such a label by Wied (see Carter 1942). It appears that in Wiesbaden no original labels are preserved. Although this is unfortunate and complicates interpretation, it does not disqualify the material as potential types, since bartered series often remained unlabeled and at the museums labels were replaced in the course of time (which of course today would be an unpardonable sin).

Wied’s work on birds is significant not only because of the huge number of species and forms described for the first time, but it also gives information on distribution and biology of numerous animals. Even today records on the biology of many organisms are completely lacking. As Berger (1995) notes, Wied’s diaries remain unstudied to date, even though they most certainly contain further biological details on many animals. Unfortunately, most of his travel journals and handwritten catalogs are privately owned and not accessible. Even so, it is important to make at least his museum specimens known to a broader scientific public, since several of Wied’s taxa are still in taxonomic transition today.

## Catalog

In our catalog we follow the systematic classification of the *Handbook of the Birds of the World* (del Hoyo et al. 1992–2011). Additionally, English and German names are given. The data of the original labels are recorded in full and unaltered, if necessary supplemented by details from the inventory catalog and the digital database of the MWNH.

Different types of printed labels are indicated as follows:

[*] Label „Naturhist. Museum Wiesbaden.“ or „Naturh. Museum Wiesbaden“, after 1917;

[**] Display label, after [*];

[***] Label „Neues Museum Wiesbaden Naturwissenschaftliche Sammlung“, after 1937.

### A. Bird specimens from Prince Maximilian of Wied-Neuwied in the MWNH collection, definitely not type material (Plate 1)

**Plate 1.**
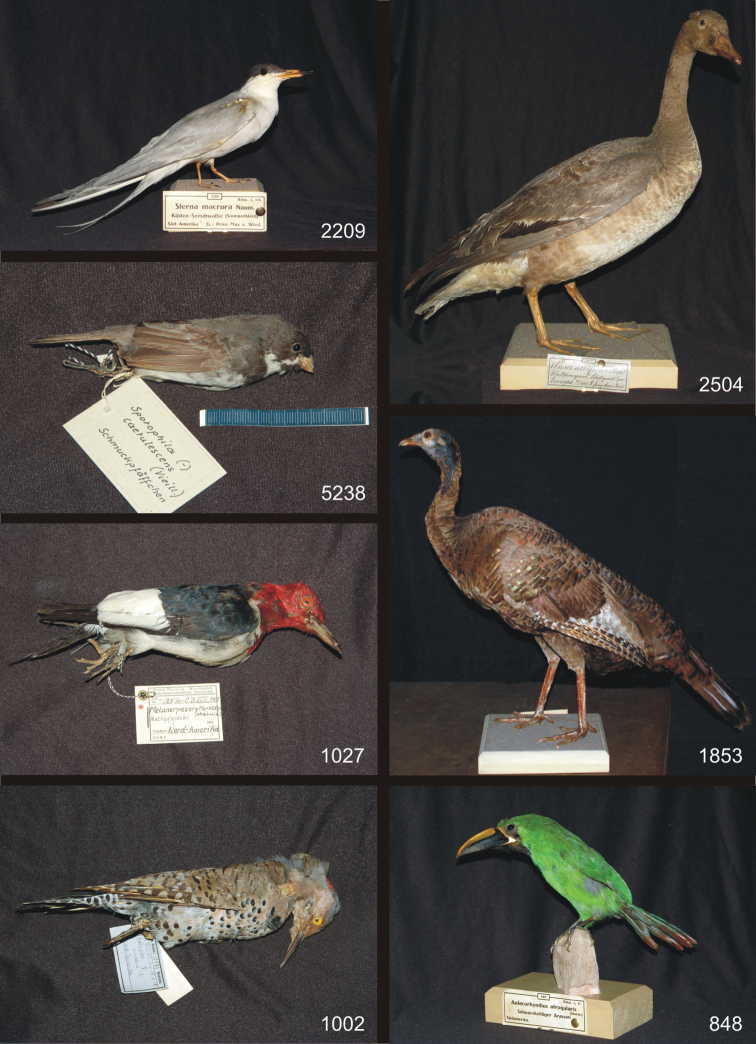
Bird specimens from Prince Maximilian of Wied-Neuwied in the MWNH collection that are definitely not type material. Clockwise from upper left: *Sterna paradisaea*, *Anser albifrons albifrons*, *Meleagris gallopavo osceola*, *Aulacorhynchus prasinus atrogularis*, *Colaptes auratus auratus*-group, *Melanerpes erythrocephalus*, *Sporophila caerulescens*.

**1. *Sterna paradisaea* Pontoppidan, 1763** (Charadriiformes – Sternidae)

Arctic Tern – Küstenseeschwalbe

Inv. nr. 2209: 1 ad., breeding plumage, mounted specimen

**Labels**: a) [*] 2209. Cat. Birds Br. Mus. XXV, 62. *Sterna macrura* Naum. Südamerika Frühjahr 1846 G.: Prinz Max v. Wied; b) [**] 2209 Rchw. 1, 116. *Sterna macrura* Naum. Küsten-Seeschwalbe (Sommerkleid) Süd-Amerika G.: Prinz Max v. Wied.

**2. *Anser albifrons albifrons* (Scopoli, 1769)** (Anseriformes – Anatidae)

Greater White-fronted Goose – Bläßgans

Inv. nr. 2504: 1 fem. juv., mounted specimen

**Labels**: a) *Anser albifrons*, Bechst. Bläßgans ♀ N.Europa; b) [*] 2504. Cat. Birds Brit. Mus. 27 pag. 92. *Anser albifrons* (Scop.) ♀ N.Europa Novbr. 1847 G.: Prinz Max v. Wied; c) [**] 2504. *Anser albifrons* (Scop.) Weißstirngans, Bläßgans ♀ juv. Europa XI/1847 G.: Prinz Max v. Wied

**3. *Meleagris gallopavo osceola* Scott, 1890** (Galliformes – Meleagrididae)

Florida Wild Turkey – Florida-Truthuhn

Inv. nr. 1853: 1 fem. ad., mounted specimen

**Labels**: a) *Meleagris Gallopavo* Nord-Amerika; b) [*] 1853 Cat. B. Br. Mus. XXII, 389/90 *Meleagris americana* Bartr. subsp. *osceola* Scott ♀ Nord-Amerika 1835. G.: Prinz Max v. Wied; c) [**] 1853 Rchw. 1, 304. *Meleagris americana* Bartr. subsp. *osceola* Scott Wildes Truthuhn Nord-Amerika S.G.: S. H. Prinz M. v. Wied

**4. *Aulacorhynchus prasinus atrogularis* (Sturm, 1841)** (Piciformes – Ramphastidae)

Emerald Toucanet – Laucharassari

Inv. nr. 848: 1 ad., mounted specimen

**Labels**: a) *Pteroglossus atrogularis* Gould. Amerika Von Prinz Max erkauft(?) [purchased from Prince Max]; b) [*] 848. Br. C. B. XIX p. 160 *Aulacorhamphus atrogularis* Sturm S.Amerika S. Prinz Max v. Wied; c) [**] 848 Rchw. 2, 37. *Aulacorhynchus atrogularis* (Sturm.) Schwarzkehliger Arassari Südamerika.; on pedestal: 1835 ans Museum [to the museum in 1835]

**5. *Colaptes auratus auratus* -group** (Linnaeus, 1758) (Piciformes – Picidae)

Yellow-shafted Flicker – Goldspecht

Inv. nr. 1002: 1 fem. ad., study skin

**Labels**: a) *Picus auratus* Nord-Amerika; b) [*] 1002 Cat. Birds Brit. Mus. 18.p.12. *Colaptes auratus* (L.) ♀ Nord-Amerika; catalog: Prinz Max von Wied, 1835

**6. *Melanerpes erythrocephalus* (Linnaeus, 1758)** (Piciformes – Picidae)

Red-headed Woodpecker – Rotkopfspecht

Inv. nr. 1027: 1 ad., study skin

**Label**: [***] Kat.Nr. 1027 Br.C.B. XVIII 145 *Melanerpes erythrocephalu* (L) Rotkopfspecht Nord-Amerika; database: erworben 1835 von Prinz Max v. Wied [purchased 1835 from Prince Max of Wied]

**7. *Sporophila caerulescens* (Vieillot, 1823)** (Passeriformes – Thraupidae)

Double-collared Seedeater – Schmuckpfäffchen

Inv. nr. 5238: 1 male ad., study skin

**Label**: [***] Kat.Nr. 5238. *Sporophila ornata* (Licht.) ♂ Brasilien leg. Prinz Max v. Wied; reverse: *Sporophila* (-)*caerulescens* (Vieill.) Schmuckpfäffchen

### B. Bird specimens from Prince Maximilian of Wied-Neuwied with potential type status (Plates 2 and 3)

**Plate 2.**
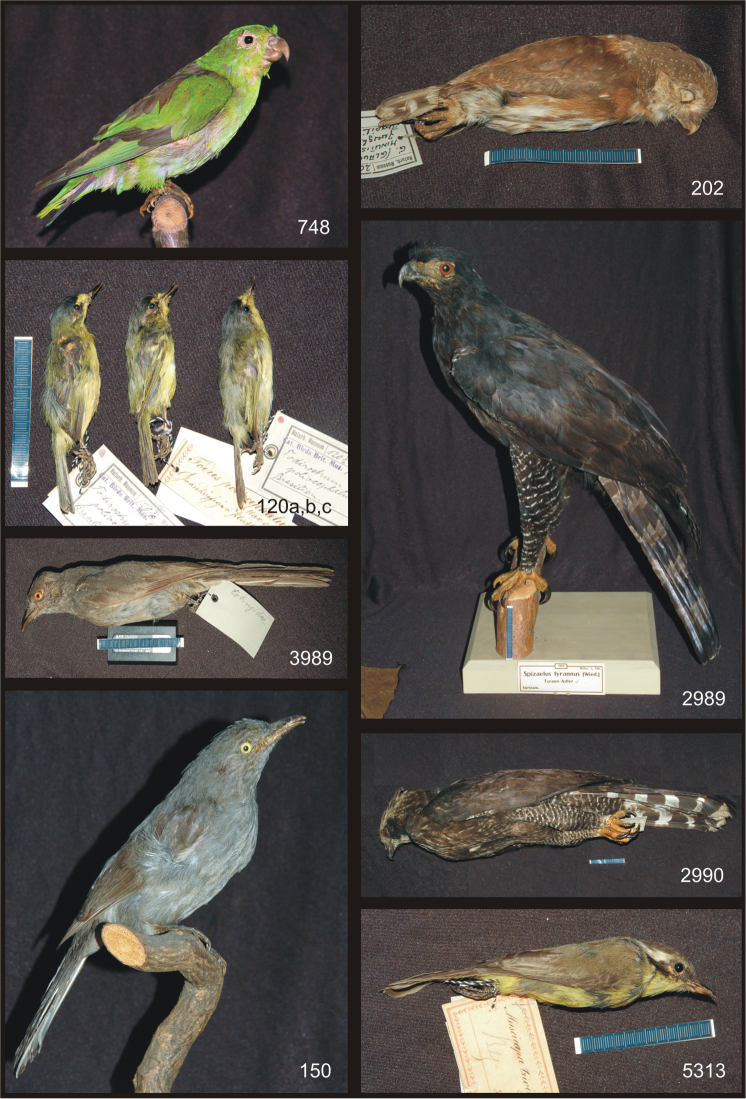
Bird specimens from Prince Maximilian of Wied-Neuwied with potential type status. Clockwise from upper left: *Touit melanonota*, *Glaucidiumminutissimum*, *Spizaetus tyrannus* (mounted), *Spizaetus tyrannus* (study skin), *Conopiastrivirgatus*, *Lipaugus vociferans* (mounted), *Lipaugus vociferans* (study skin), *Todirostrum poliocephalum*.

**Plate 3.**
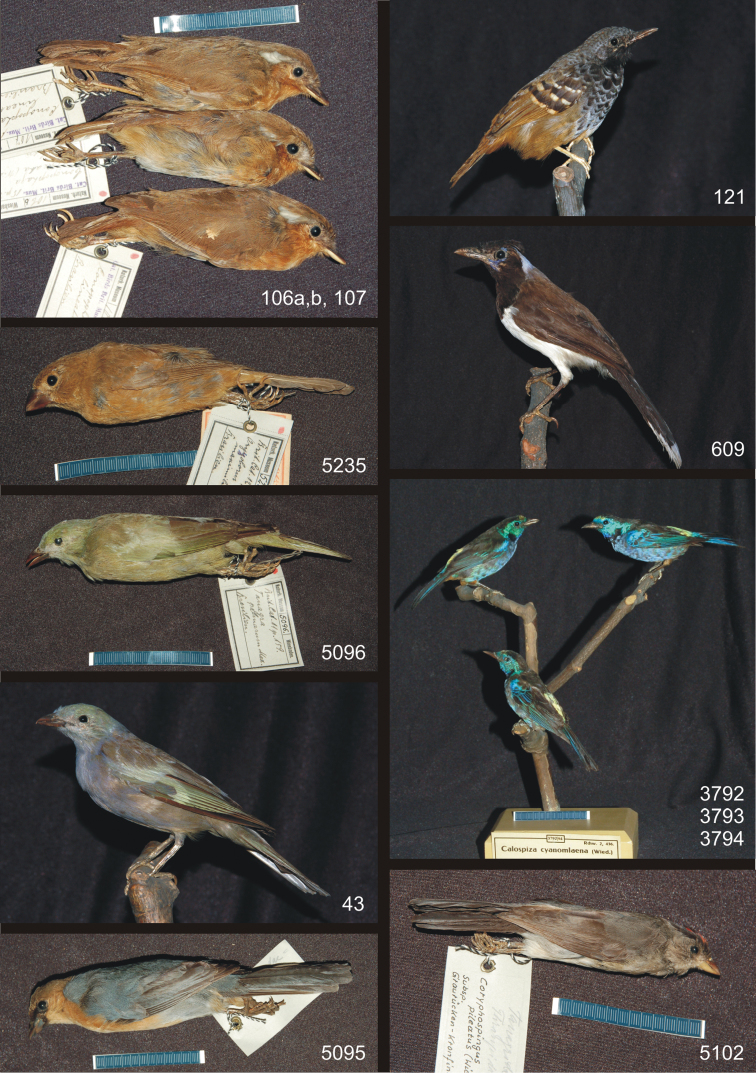
Bird specimens from Prince Maximilian of Wied-Neuwied with potential type status, continued. Clockwise from upper left: *Conopophaga lineata*, *Myrmeciza ruficauda*, *Cyanocorax cyanopogon*, *Tangaravelia cyanomelas*, *Coryphospingus pileatus*, *Schistochlamys ruficapillus capistratus*, *Thraupis palmarum palmarum* (mounted), *Thraupis palmarum palmarum* (study skin), *Oryzoborus maximiliani*.

**1. *Touit melanonota* (Wied, 1820)** (Psittaciformes – Psittacidae)

Brown-backed Parrotlet – Braunrückenpapagei

Inv. nr. 748: 1 ad., mounted specimen

**Syn.**: *Psittacus melanonotus* Wied, 1820: 275; *Urochroma melanota* (Stephens, 1826)

**Labels**: a) *Psittacus melanotus* Brasilien; b) [*] 748 Br.C.B. XX. p. 352. *Urochroma wiedi* Allen Brasilien.; c) [**] 748 Rchw. 1, 484. *Urochroma melanota* (Lcht.) Wied Schwarzrückiger Zwergpapagei Brasilien.; catalog: Prinz Max v. Wied, 1835

**Remarks**: According to the original description, Wied saw several animals and noted that this new species was displayed at the Berlin museum under the name *Psittacus melanonotus*. Berger (1995: 291 with pictures from the AMNH, inv. nr. 6302) states that there is one type specimen in New York. According to M. LeCroy (AMNH, pers. comm.), this type was listed by Allen (1889: 264-265) but, when included in the genus *Psittacus*, Wied’s name was preoccupied by *Psittacus melanotus* Shaw, 1804. Allen provided a replacement name, *Urochroma wiedi*. Wied’s form is now placed in the genus *Touit* (del Hoyo et al. 1997, Vol. IV: 456) and his species name, *melanonotus*, is being used again. *Urochroma wiedi* Allen 1889 is a synonym sharing the same type/types. Greenway (1978: 86) was apparently in error in listing AMNH 6302 as a holotype, as Allen (1889: 265) noted that there was at least one additional syntype and perhaps others. Presently there is nothing to be said against the assumption that the specimen at the MWNH is also a syntype.

**2. *Glaucidium minutissimum* (Wied, 1830)** (Strigiformes – Strigidae)

Least Pygmy-owl – Kleinstzwergkauz

Inv. nr. 202: 1 ad., study skin

**Syn.**: *Strix minutissima* Wied, 1830: 242

**Label**: [*] 202 *Glaucidium (Glaucidium) minutissimum* (Wied) Zwergkauz Brasilien

**Remarks**: According to the original description, Wied examined males and females, but does not give any information about the quantities or the whereabouts. Allen (1889: 266) and Greenway (1978: 126) report two syntypes of *Strix minutissima* at the AMNH with the inventory numbers 6345 (male) and 6345 bis (female). The MWNH specimen might belong to the original type series, although there is no proof.

**3. *Spizaetus tyrannus* (Wied, 1820)** (Falconiformes – Accipitridae)

Black Hawk-eagle – Tyrannenadler

I. Inv. nr. 2989: 1 male ad., mounted specimen

II. Inv. nr. 2990: 1 juv., study skin

**Syn.**: *Falco tyrannus* Wied, 1820: 360

**Labels**: I. a) No. 109 *Falco Tyrannus* Pr. Max Temm. Pl. col. 73. Seite 61 Cuv. Seite 384. Iris gelb.; b) [*] 2989. Brit. Cat. I. 264 *Spizaetus tyrannus* Wied. ♂ Surinam; c) [**] 2989 Rchw. 1, 386. *Spizaetus tyrannus* (Wied.) Tyrann-Adler ♂ Surinam.; II. [***] Kat.Nr. 2990 R. I. 386 *Spizaetus tyrannus* (Wied) Tyrann-Adler Surinam

**Remarks**: According to the original description, Wied had at least one male at his hands. The AMNH has a specimen (inv. nr. 6381) that Greenway (1973: 270) called a lectotype. Of the specimens at the MWNH, the more recent labels give “Surinam” as the country of origin, but the older label contains a reference to “Pr. Max”. Wied may have had specimens from Surinam in his collection that he did not collect himself (M. LeCroy, pers. comm.). Thus it appears quite possible that these two specimens are type material.

**4. *Conopias trivirgatus* (Wied, 1831)** (Passeriformes – Tyrannidae)

Three-striped Flycatcher – Olivbrust-Maskentyrann

Inv. nr. 5313: 1 ad., study skin

**Syn.**: *Muscicapa trivirgata* Wied, 1831: 871

**Labels**: a) *Muscicapa trivirgata* unserer(?) Beiträge; b) *Muscicapa trivirgata* M. v. Wied, Brasilien; c) [*] 5313. Cat. Birds Brit. Mus. *Conopias (Conopias) trivirgata* (Wied) Subsp. Dreistreifentyrann Brasilien

**Remarks**: According to the original description, Wied examined one female from Bahia. Greenway (1987: 34) listed a female holotype at the AMNH under the number 4926. Allen (1889: 234) noted that “Femina” was not written in Wied’s catalog, but AMNH 4926 is sexed as a female. Since Wied himself only mentions one female, the specimen at the AMNH probably is the holotype.

**5. *Lipaugus vociferans* (Wied, 1820)** (Passeriformes – Cotingidae)

Screaming Piha – Tiefland-Graupiha

I. Inv. nr. 150: 1 ad., mounted specimen

II. Inv. nr. 3989: 1 fem. ad., study skin

**Syn.**: *Muscicapa vociferans* Wied, 1820: 242

**Labels**: I. a) [*] 150 Cat. Birds Brit. Mus. 14 p.352. *Lathria cinerea* (Vieill.) Surinam; b) [**] 150 Rchw. 2, 191. *Lipaugus cinereus* (Vieill.) Surinam.; II. [*] 3989. R. II. 191. *Lipaugus cinereus* (Vieill.) Brasilien G.: Geschw. Brambeer.; reverse: Cotingidae

**Remarks**: In the original description Wied does not state the quantity of examined specimens. According to him, this new species was displayed at the Berlin museum under the name *Muscicapa ampelina*. Greenway (1987: 42) listed two syntypes in the AMNH collection, one female with the number 5099 and one male with 5098. Allen (1889: 239) misquotes the numbers as 5198 and 5199. Due to the reference “G: Brambeer” the study skin (inv. nr. 3989) at the MWNH is probably not a type specimen. The mounted specimen (inv. nr. 150) might be type material, since Wied may have used specimens that he did not collect himself (see above).

**6. *Todirostrum poliocephalum* (Wied, 1831)** (Passeriformes – Tyrannidae)

Grey-headed Tody-flycatcher – Gelbzügel-Todityrann

I. Inv. nr. 120 a: 1 ad., study skin

II. Inv. nr. 120 b: 1 ad., study skin

III. Inv. nr. 120 c: 1 ad., study skin

**Syn.**: *Todus poliocephalus* Wied, 1831: 964

**Lab els**: I. a) *Todus poliocephalus*, Pr. Max Grauköpfiger Plattschnabel Brasilien; b) [*] 120 3/a Cat. Birds Brit. Mus. 14 p. 71. *Todirostrum poliocephalum* (Wied) Brasilien.; II. a) *Todus poliocephalus* Max v. Wied. Grauköpfiger Plattschnabel Brasilien; b) [*] 120 3/b Cat. Birds Brit. Mus. 14. 71 *Todirostrum poliocephalum* (Wied.) Brasilien; III. a) *Todus policephalus*, Max v. [abgeschnitten] Grauköpfiger Plattschnabel Brasilien; b) [*] 120 3/c Cat. Birds Brit. Mus. 14 p. 71 *Todirostrum poliocephalum* (Wied) Brasilien.; reverse of b) (all): Tyrannidae

**Remarks**: In the original description Wied examined males and females, but did not note the quantities and whereabouts. Allen (1889: 228) listed a male (nr. 6790) and a female syntype (nr. 6791) in the AMNH collection. The original Wied label pasted to the reverse of the AMNH label on the male indicates both sexes and originally served for both specimens. The three MWNH specimens might well stem from Wied and must hence be regarded as potential type material.

**7. *Conopophaga lineata* (Wied, 1831)** (Passeriformes – Conopophagidae)

Rufous Gnateater – Rotkehl-Mückenfresser

I. Inv. nr. 106 a: 1 ad., study skin

II. Inv. nr. 106 b: 1 ad., study skin

III. Inv. nr. 107: 1 ad., study skin

**Syn.**: *Myiagrus lineatus* Wied, 1831: 1046

**Labels**: I. [*] 106a. Cat. Birds Brit. Mus. 15. p. 333 *Conopophaga lineata* (Wied) Brasilien; II. [*] 106b. Cat. Birds Brit. Mus. 15 p. 333. *Conopophaga lineata* (Wied.) Brasilien; III. [*] 107 Cat. Birds Brit. Mus. 15 p. 333. *Conopophaga lineata* (Wied.) Brasilien

**Remarks**: In the original description Wied mentions only one individual. The AMNH has one female holotype (Nr. 6777) listed by Allen (1889: 256; see also LeCroy and Sloss 2000: 65). Since Wied referred to only one individual, and as we have no proof that the MWNH specimens originate from him, they are probably not type material.

**8. *Myrmeciza ruficauda* (Wied, 1831)** (Passeriformes – Thamnophilidae)

Scalloped Antbird – Nördlicher Schuppenameisenvogel

Inv. nr. 121: 1 ad., mounted specimen

**Syn.**: *Myiothera ruficauda* Wied, 1831: 1060

**Labels**: a) *Formicivora loricata* ♀ Swains. Bahia; b) *Formicivora* ♂. Bahia. 3. Gust. Schneider, Basel.; c) [*] 121. Cat. Birds Brit. Mus. 15 p. 281. *Myrmeciza ruficauda* Wied Bahia S.: G. Schneider, Basel.; d) [**] 121 Rchw. 2, 231. *Myrmeciza ruficauda* Wied. Bahia.

**Remarks**: In the original description Wied examined males and females, but does not specify the quantities and whereabouts. There are four syntypes at the AMNH: males nr. 5388 and 6829, juvenile male nr. 5386 and female nr. 5385 (Allen 1889: 254; LeCroy and Sloss 2000: 56-57). Because of its origin the specimen at the MWNH can be ruled out as type material.

**9. *Cyanocorax cyanopogon* (Wied, 1821)** (Passeriformes – Corvidae)

White-naped Jay – Weißnacken-Blaurabe

Inv. nr. 609: 1 ad., mounted specimen

**Syn.**: *Corvus cyanopogon* Wied, 1821: 137

**Labels**: a) *Corvus cyanopogon* Brasilien; later altered: *Cyanocorax Corvus cyanopogon* (Max Neuwied) III, 123.; b) [*] 609 Br. C. B. III p. 123 *Cyanocorax cyanopogon* (Neuwied) Brasilien.

**Remarks**: In the original description Wied had several individuals at hand. Allen (1889: 227) lists two syntypes (juvenile female nr. 6773 and male nr. 6774) at the AMNH. It cannot be excluded that the mounted specimen at the MWNH is also type material.

**10. *Tangara velia cyanomelas* Wied, 1830** (Passeriformes – Thraupidae)

Silvery-breasted Tanager – Rotbauchtangare; group of 3 individuals

I. Inv. nr. 3792: 1 male ad., mounted specimen

II. Inv. nr. 3793: 1 fem. ad., mounted specimen

III. Inv. nr. 3794: 1 fem. ad., mounted specimen

**Syn.**: *Tangara cyanomelas* Wied, 1830: 453

**Labels**: a) *Tanagrella velia* ♂♀? Gmel Bahia; b) [*] 3792/94 Brit.Cat.11 p. 88. *Tanagrella cyanomelaena* (Wied.) ♂♀♀ Bahia; c) [**] 3792/94 Rchw. 2, 436. *Calospiza cyanomlaena* (Wied.) 1 ♂, 2 ♀ Bahia, Brasilien.

**Remarks**: In the original description Wied mentions several males, while the female was unknown to him. According to Allen (1889: 218) and LeCroy (2012) there are no types at the AMNH. If no other material turns up, the specimens at the MWNH must be classified as potential types. First of all, they need to be sexed, as Wied had no females at hand. The sexes assigned on the labels were possibly inferred from the individuals’ positions (male on top, females below) by a curator. On first glance the three specimens are indistinguishable and must be compared to a series.

**11. *Coryphospingus pileatus* (Wied, 1821)** (Passeriformes – Thraupidae)

Pileated Finch – Graurückenkronfink

Inv. nr. 5102: 1 male ad., study skin

**Syn.**: *Fringilla pileata* Wied, 1821: 160

**Label**:[*] 5102. Cat. Birds Brit. Mus. *Tanagra cristatella* Spix. ♂ Brasilien; reverse: Tanagridae = Thraupidae *Coryphospingus pileatus* (Wied) Subsp. Graurücken-Kronfink

**Remarks**: In the original description Wied described the male without giving details on quantity or whereabouts of the specimens. According to Allen (1889: 225) and LeCroy (2012) there are three male syntypes at the AMNH (nr. 4618, 4619, and 4621). Comparison of the skins at the AMNH and the MWNH should help to decide whether the specimen in Wiesbaden, with identical or similar taxidermy, belongs to the type series.

**12. *Schistochlamys ruficapillus capistratus* (Wied, 1821)** (Passeriformes – Thraupidae)

Cinnamon Tanager – Gimpeltangare

Inv. nr. 5095: 1 ad., study skin

**Syn.**: *Tanagra capistrata* Wied, 1821: 179

**Label**: [*] 5095. Brit.Cat.11 p. 301. *Schistochlamys capistratus* (Max) Brasilien.; reverse: Tanagridae = Thraupidae

**Remarks**: In the original description, and also in 1831 (p. 500), Wied examined males and females, but did not note specifics on quantities and whereabouts. According to Allen (1889: 222) and LeCroy (2012) the AMNH has only one male syntype (nr. 6861). Since at least the female is still missing, and also the number of individuals remains unclear, the specimen in Wiesbaden may well be classified as potential type material. In this species the sexes look alike, so it will be difficult to determine the specimen’s sex.

**13. *Thraupis palmarum palmarum* (Wied, 1821)** (Passeriformes – Thraupidae)

Palm Tanager – Palmentangare

I. Inv. nr. 43: 1 ad., mounted specimen

II. Inv. nr. 5096: 1 ad., study skin

**Syn.**: *Tanagra palmarum* Wied, 1821: 76

**Labels**: I. a) *Tanagra palmarum* Mexiko; b) [*] 43 Brit.Cat. 11 p. 159. *Tanagra palmarum* Max. Brasilien. Mexiko auf alt/Schauetik.; c) [**] 43 Rchw. 2, 435. *Tanagra palmarum* Max. Brasilien.; II. [*] 5096. Brit.Cat.11 p.159. *Tanagra palmarum* Max. Brasilien.

**Remarks**: In the original description Wied refers to both sexes, but does not specify the quantity and whereabouts of the examined specimens. Allen (1889: 219) notes a male syntype at the AMNH (nr. 6765), which LeCroy (2012) confirms. Both specimens at the MWNH are unsexed. The oldest label of nr. 43 gives Mexico as origin, while the more recent ones point out a mistake. The study skin is a formerly mounted specimen and does not have an old label. Whether or not these specimens are type material cannot be determined.

**14. *Oryzoborus maximiliani* Cabanis, 1851** (Passeriformes – Thraupidae)

Great-billed Seed-finch – Dickschnabel-Reisknacker

Inv. nr. 5235: 1 fem. ad., study skin

**Syn.**: *Oryzoborus crassirostris* Wied, 1830: 564, preocc. *Oryzoborus crassirostris* (Gmelin, 1789)

**Labels**: a) *Fringilla crassirostris*, Max v. Wied *Pyrrhula crassirost*. ♀ Brasilien; b)[*] 5235. Brit.Cat.12 p. 78 *Oryzoborus maximiliani* Cab. ♀ Brasilien.

**Remarks**: Unfortunately we could not obtain the original description. Cabanis (1851: 151) replaced the homonym. According to Allen (1889: 222) and LeCroy (2012) there is no specimen at the AMNH. If no other specimen turns up, the study skin at the MWNH must be regarded as type material.

## Conclusion

In conclusion, it can be assumed that 17 individuals in 11 species of birds in the Wiesbaden collection are potential type specimens that should be considered in future taxonomic work. Close examination, comparison with other material, or even genetic tests will be necessary to make a final decision on the specimens’ type status.
